# Systematic reviews addressing identified health policy priorities in Eastern Mediterranean countries: a situational analysis

**DOI:** 10.1186/1478-4505-12-48

**Published:** 2014-08-20

**Authors:** Fadi El-Jardali, Elie A Akl, Lama Bou Karroum, Ola Kdouh, Chaza Akik, Racha Fadlallah, Rawan Hammoud

**Affiliations:** 1 Department of Health Management and Policy, American University of Beirut, PO Box 11-0236, Riad El Solh, Beirut 1107 2020, Lebanon; 2 Center for Systematic Reviews of Health Policy and Systems Research (SPARK), American University of Beirut, PO Box 11-0236, Riad El Solh, Beirut 1107 2020, Lebanon; 3 Research, Advocacy and Public Policy-making Program, Issam Fares Institute for Public Policy and International Affairs, American University of Beirut, PO Box 11-0236, Riad El Solh, Beirut 1107 2020, Lebanon; 4 Department of Clinical Epidemiology and Biostatistics, McMaster University, CRL-209, 1280 Main St. West, Hamilton, Ontario L8S 4 K1, Canada; 5 Department of Internal Medicine, American University of Beirut, PO Box 11-0236, Riad El Solh, Beirut 1107 2020, Lebanon; 6 Department of Population Health, London School of Hygiene and Tropical Medicine, Keppel St, Bloomsbury, London WC1E 7HT, UK

**Keywords:** Eastern Mediterranean region, Health policy and systems research, Policy priorities, Systematic reviews

## Abstract

**Background:**

Systematic reviews can offer policymakers and stakeholders concise, transparent, and relevant evidence pertaining to pressing policy priorities to help inform the decision-making process. The production and the use of systematic reviews are specifically limited in the Eastern Mediterranean region. The extent to which published systematic reviews address policy priorities in the region is still unknown. This situational analysis exercise aims at assessing the extent to which published systematic reviews address policy priorities identified by policymakers and stakeholders in Eastern Mediterranean region countries. It also provides an overview about the state of systematic review production in the region and identifies knowledge gaps.

**Methods:**

We conducted a systematic search of the Health System Evidence database to identify published systematic reviews on policy-relevant priorities pertaining to the following themes: human resources for health, health financing, the role of the non-state sector, and access to medicine. Priorities were identified from two priority-setting exercises conducted in the region. We described the distribution of these systematic reviews across themes, sub-themes, authors’ affiliations, and countries where included primary studies were conducted.

**Results:**

Out of the 1,045 systematic reviews identified in Health System Evidence on selected themes, a total of 200 systematic reviews (19.1%) addressed the priorities from the Eastern Mediterranean region. The theme with the largest number of systematic reviews included was human resources for health (115) followed by health financing (33), access to medicine (27), and role of the non-state sector (25). Authors based in the region produced only three systematic reviews addressing regional priorities (1.5%). Furthermore, no systematic review focused on the Eastern Mediterranean region. Primary studies from the region had limited contribution to systematic reviews; 17 systematic reviews (8.5%) included primary studies conducted in the region.

**Conclusions:**

There are still gaps in the production of systematic reviews addressing policymakers’ and stakeholders’ priorities in the Eastern Mediterranean region. Efforts should be directed towards better aligning systematic review production with policy needs and priorities. Study findings can inform the agendas of researchers, research institutions, and international funding agencies of priority areas where systematic reviews are required.

## Background

Evidence-informed policies can strengthen national health systems and improve health outcomes. Specifically, evidence assists policymakers and stakeholders in identifying priorities, providing a broad choice of policy options to address priorities, informing policy formulation and implementation, and setting the stage for evaluating the outcomes of policies [1,2]. Evidence that can inform policy decisions can be derived from sources such as research, expert opinion, grey reports, and local health systems indicators. For the sake of this study we are only looking at research evidence derived from systematic reviews (SRs).

SRs allow the identification and synthesis of relevant and up-to-date research evidence. They can offer policymakers and stakeholders concise, transparent, and relevant evidence pertaining to pressing policy priorities to help inform the decision-making process. SRs are reviews of the literature characterized by five main components: explicit questions, search strategy, eligibility criteria, critical appraisal of the quality of the included studies, and a clear and transparent method of synthesis [3]. They constitute a more appropriate source of research evidence than individual studies. First, the probability of being misled by research evidence is lower with a SR than with an individual study. Second, confidence in an intervention’s effectiveness is higher with a SR than with an individual study. Third, SRs provide a summary of the best quality studies available so drawing on an existing SR constitutes a more efficient use of time. Fourth, a SR can be more constructively contested than an individual study. Fifth, SRs summarize the findings of studies conducted in different settings so they make it easier for users to assess the applicability of a certain option [3,4]. Even in the absence of high quality individual studies to inform SRs, the latter can serve to understand the quality of literature overall, and as baseline assessment and synthesis of existing research evidence on a particular topic.

The production and the use of SRs are specifically limited in the Eastern Mediterranean region (EMR). A study conducted by Law et al. assessing the profile of SR production in 41 low- and middle-income countries (LMICs) found that the EMR is among the lowest in terms of SR production [5]. Only 10% of the total studies included are produced by a corresponding author based in the EMR. The same study found that the EMR also ranked the lowest in terms of being the target of a SR in comparison to Asia, Africa, and the Americas. In an exercise to assess the climate for use of evidence in policy conducted in 11 countries in the EMR, around 65% of respondents indicated that SRs on high priority issues were rarely disseminated to policymakers [6]. Policymakers from six LMICs including countries from the EMR, highlighted the need for better packaging of research results to assist in evidence-informed policymaking [7]. A more extensive survey of policymakers in 12 EMR countries revealed the need for easier access to information [8].

Providing policymakers with policy-relevant research and user-friendly formats and engaging them in research priority-setting exercises would increase the prospects of evidence utilization by policymakers [3,9,10]. Over the last five years, two systematic priority-setting exercises to identify policy-relevant priorities were conducted in the EMR. The first priority-setting exercise derived global priorities on human resources for health (HRH), health financing, and the role of the non-state sector based on country-level priority research [11]. The second priority setting was a subset of the first and was conducted in nine countries of the EMR and identified policy priorities pertaining to HRH, health financing, and the role of the non-state sector [12]. The countries included in the study are Algeria, Lebanon, Jordan, Egypt, Morocco, Palestine, Syria, Yemen, and Tunisia. The third priority-setting exercise was conducted in three countries of the region (Lebanon, Iran, and Pakistan) and identified policy priorities in the area of access to medicine (ATM) [13].

The three exercises elicited the priorities voiced by policymakers and stakeholders from the public sector, academia, the non-state sector, and health professional associations in addition to pharmaceutical companies and insurance organizations for the ATM theme (Refer to Table 1 for identified policy relevant priorities matched to the second and third exercises which were conducted in the EMR).

**Table 1:** **Systematic reviews matched with the policy-relevant priorities identified in El-Jardali et al. **[12]**and Rashidian et al. **[13]**by theme**

**Rank**	**Policy-relevant priorities**	**N (%)**
	**Theme 1: Human Resources for Health (HRH)**	**115**
**1**	Means to develop HRH information systems in ministries of health and national observatories	0 (0.0)
**2**	Gaps in existing education and training programs	0 (0.0)
**3**	Information on patient satisfaction	1 (0.9)
**4**	Accurate estimates and needs in numbers and specialties (mapping)	4 (3.5)
**5**	Ways that can enable education and training programs to meet the population health needs	30 (26.1)
**6**	Methods to measure HRH performance and productivity	0 (0.0)
**7**	Develop simulation models for HRH planning	30 (26.1)
**8**	Elements of performance evaluation	7 (6.1)
**9**	Develop incentive mechanisms to better manage the existing stock of HRH	18 (15.7)
**10**	Ways to improve staff satisfaction	25 (21.7)
	**Theme 2: Health Financing**	**33**
**1**	Elements of an equitable health financing system	13 (39.3)
**2**	Household ability to pay for healthcare	16 (48.4)
**3**	Linking population health needs to health spending	2 (6.1)
**4**	Role of the social health insurance system in guaranteeing equity	1 (3.0)
**5**	Identifying best practices to develop and implement a national social health insurance system	9 (27.2)
**6**	Clarifying functions and coordination processes between ministries (for example, the ministries of health and of finance) to improve health system financing and quality of services	0 (0.0)
**7**	Means to track financial resources invested in health care to ensure value for money	0 (0.0)
**8**	Accurate estimation of the health expenditure from the public and the private sectors including out-of-pocket expenditure	0 (0.0)
**9**	Population health status and needs	0 (0.0)
	**Theme 3: Role of the Non-State Sector**	**25**
**1**	Ways to regulate and monitor the quality of care in the private sector	16 (64.0)
**2**	Ways to optimize the use of the existing resources of the non-state sector to meet health system objectives	4 (16.0)
**3**	Ways for the public and private sectors to complement their service delivery	4 (16.0)
**4**	Areas where the state and civil society groups can complement each other	0 (0.0)
**5**	National database on the non-state sector	0 (0.0)
**6**	Foundation/elements for building strong public-private partnerships	1 (4.0)
**7**	Accreditation standards for private sector	0 (0.0)
**8**	Ways to develop effective contracting mechanisms with the private and other non-state sectors	3 (12.0)
**9**	National plan for the contribution of the non-state sector	0 (0.0)
**10**	Measuring client satisfaction	0 (0.0)
**11**	Defining the role and responsibility of the non-state sector	0 (0.0)
**12**	Scope, resources, and kind of services provided by the non-state sector	0 (0.0)
	**Theme 4: Access to Medicine**	**27**
**1**	Evaluation the role of pharmaceutical companies on prescribing and drug use patterns	5 (18.5)
**2**	Identifying effective continuous education methods for physicians to improve drug use patterns and access to medicines	8 (29.6)
**3**	What happens at the dispensary? Dispensing medicines or delivering primary health care?	2 (7.4)
**4**	Identifying effective methods on improving public knowledge and awareness about drug use	3 (11.1)
**5**	Consumer demand, health-seeking preferences, willingness to pay, and enhancing patient role in accountability	0 (0.0)
**6**	Assessing the procedures and regulations for adding medicines to the national drug list (formulary) and identifying improvement models	0 (0.0)
**7**	Adherence to generics in primary health care and dispensaries	1 (3.7)
**8**	Attitudes of physicians and of the public towards generic substitution and the opportunities for implementing relevant policies	0 (0.0)
**9**	Pricing policies to improve access to essential generics and contain prices of excessively priced originator brands	0 (0.0)
**10**	Evaluation of the effect of the ‘single item importing’ policy on final cost of medicines, quality and access, and health system expenditure	0 (0.0)
**11**	Evaluation of the process of adding medicines to the insurance organizations' list of medicines covered	0 (0.0)
**12**	Alternative financing mechanisms to supplement public sector provision	12 (44.4)
**13**	Optimal mix of pricing regulations to reduce expenditure burden on households	1 (3.7)
**14**	Assessment of quality of medicines on the market and role of counterfeit medicines and black market	0 (0.0)
**15**	Improving logistics and human resource management in the public sector for improving drug access	0 (0.0)
**16**	Evaluation of the role of civil society organizations and non-governmental organizations in improving access to medicines especially for the poor, vulnerable groups, and hard-to-reach populations	0 (0.0)
**17**	Mapping and assessment of private sector including of qualified providers, informal providers, shadow pharmacies, and traditional healers	0 (0.0)

**Figure 1:**
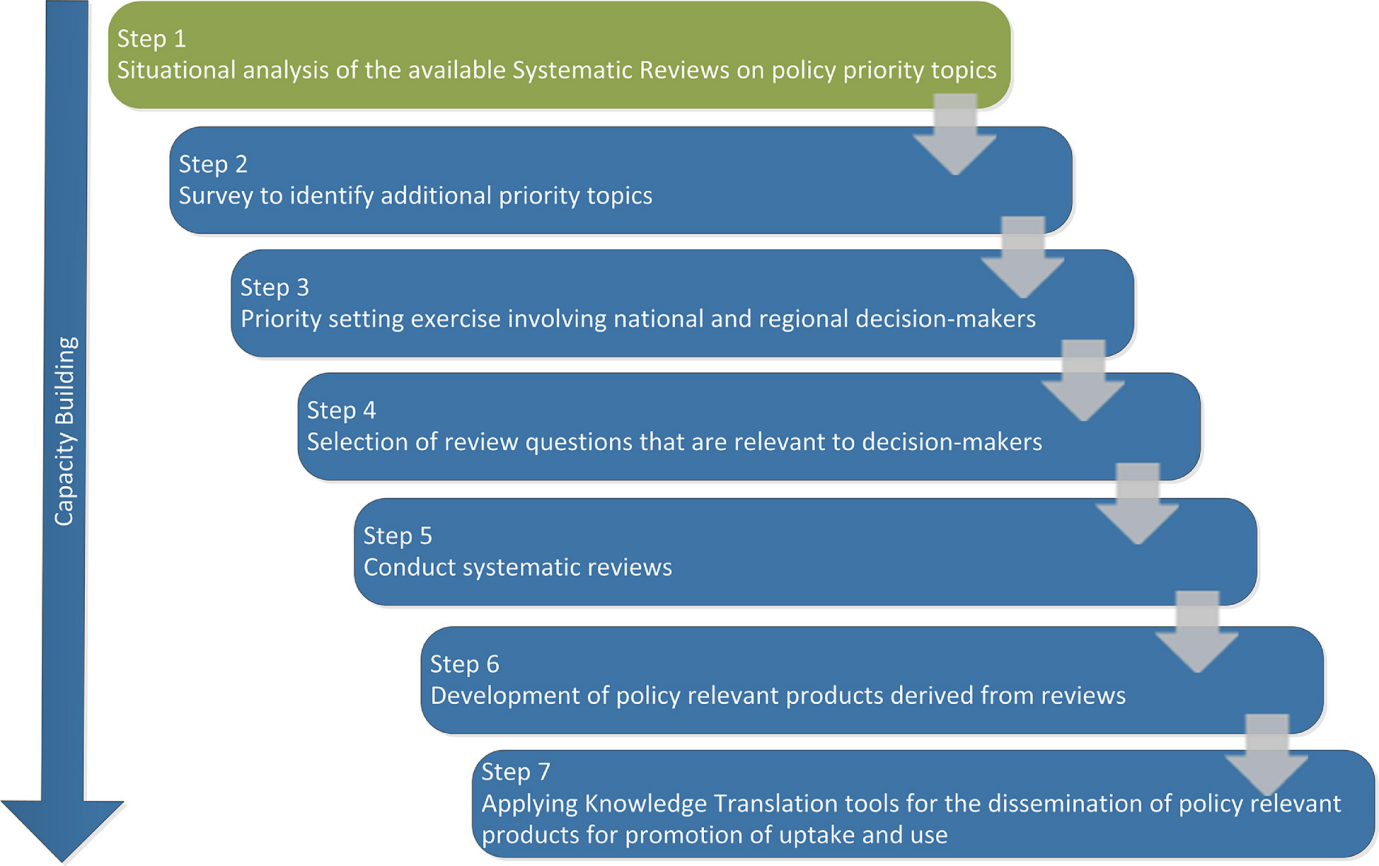
Planned activities of the WHO Center for systematic reviews on health policy and systems research.

The four themes: HRH, health financing, the role of the non-state sector, and the ATM were selected given their central role in improving health systems and health outcomes. In fact, the WHO 2007 report on strengthening health systems presents the six building blocks of health system strengthening, which include health financing, HRH, and health delivery (including non-state delivery) as essential components of their framework [14]. Countries in the EMR are still facing challenges regarding the four themes. The EMR has the second lowest HRH density worldwide; and the nine LMICs of the region lack sound HRH policies, planning, and efficient management as well as the capacity of educational and training programs [12]. Moreover, in many EMR countries, the state is not the sole provider of health care services with an increasing role for the non-state sector [12], and a number of health financing issues that are common to this region include poor resource allocation, poor public-private partnerships, and a lack of policies for financial sustainability [15]. In terms of the fourth priority topic, ATM was considered by the Alliance for Health Systems and Policy Research as a thematic area that requires new research and analysis [16]. In many EMR countries, there is poor access to appropriate medicines and limited contextual evidence on ATM to assist in policy development [13]. The extent to which the identified policy priorities are addressed by published SRs is still unknown. To our knowledge, no previous research was conducted to map out which priorities are already addressed by SRs.In a step to enhance production and use of SRs in the EMR, the Alliance for Health Policy and Systems Research at the World Health Organization (WHO) provided funding to establish the Center for Systematic Reviews on Health Policy and Systems Research at the American University of Beirut. The Center will develop SRs to address high policy priority issues in the EMR and will also build individual and institutional capacity of young researchers in conducting SRs on Health Policy and Systems Research. Figure 1 illustrates the planned activities of the Center. The first step is to conduct a situational analysis exercise to identify SRs addressing policy priorities identified by policymakers and stakeholders in EMR countries. This situational analysis exercise aims at i) assessing the extent to which published SRs address policy priorities identified by policymakers’ and stakeholders in EMR countries; ii) providing an overview about the state of SR production in the region; and iii) identifying knowledge gaps.

## Methods

**Figure 2:**
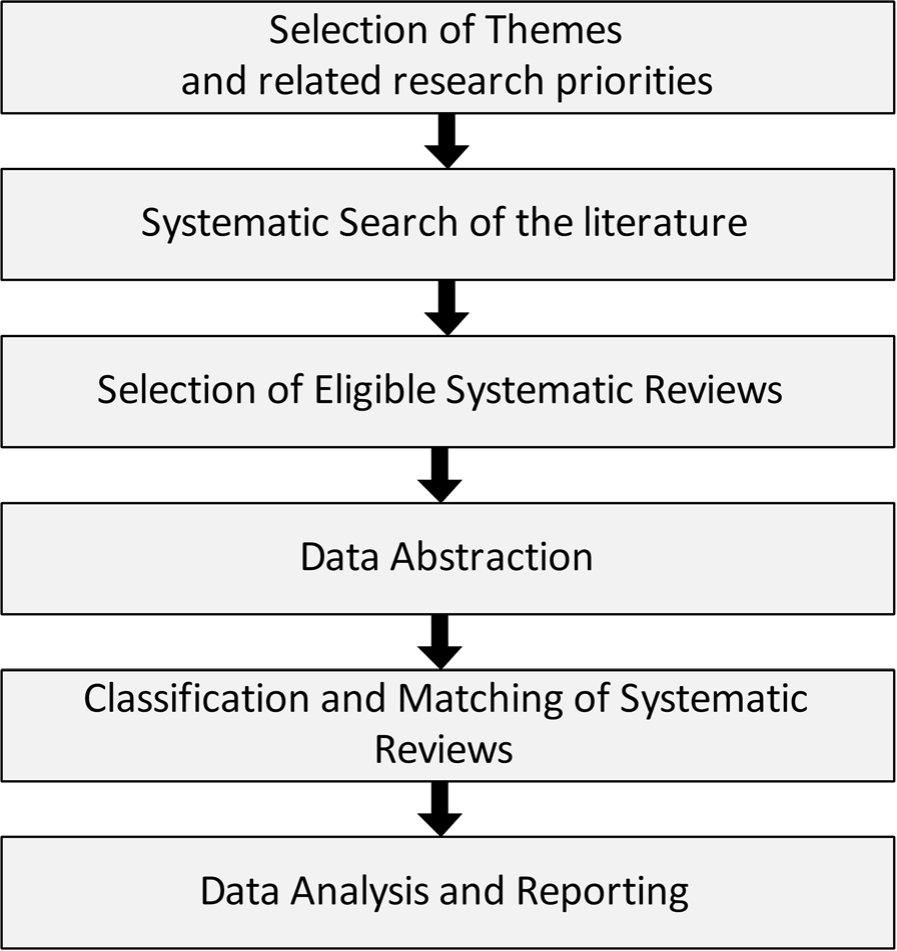
Phases of the study methodology.

A systematic search was conducted to identify published SRs on policy-relevant research priorities pertaining to the following themes: HRH, health financing, the role of the non-state sector, and ATM, as identified in the two priority-setting exercises conducted in the region [12,13]. Although the priority-setting exercises focused on LMICs, given the low proportion of high-income countries in the region, we will consider those priorities to be representative of the EMR. Further, we described the distribution of these SRs across themes, sub-themes, authors’ affiliations, and countries where included primary studies were conducted. The details of the methodology are described in Figure 2.

### Eligibility criteria

We included publications that met the following criteria:

• Described by its authors as a SR and/or a meta-analysis or following the methodology of a SR and/or a meta-analysis.

• Included a search strategy of at least one electronic database.

• Compared at least two interventions or an intervention versus no intervention.

• Addressed or can provide information to address at least one of the identified policy-relevant research priorities pertaining to the HRH, health financing, role of the non-state sector, and ATM [12,13].

We did not exclude SRs based on language or date of publication. We excluded non-peer reviewed publications and overviews of SRs. We also excluded SRs being planned and those in progress.

### Search strategy

We searched the Health Systems Evidence (HSE) database in June 2013. HSE is a comprehensive and continuously updated database of SRs for health systems and policy topics (available at healthsystemsevidence.org). HSE is an initiative of the McMaster Health Forum targeting health systems policymakers and stakeholders, among others. The content of HSE is drawn from major sources such as the Cochrane Library for SRs of effects and protocols for such reviews, the Economic Evaluation Database for economic evaluations, and the Health Policy Monitor for health reform descriptions.

For all themes, we used the relevant search terms included in the HSE database taxonomy. For the role of the non-state sector and ATM themes, we complemented the search with terms from the frameworks presented under the section “Classification of systematic reviews” and the previous priority setting exercises. Additional file 1 lists the search strategies per theme.

### Selection process

Search results were imported into Excel sheets and duplicates were removed. We conducted the selection process in three stages: title screening, abstract screening, and full-text screening. Two reviewers screened in duplicate and independently. In the first stage, titles were screened to determine relevance. Titles were considered eligible to pass to the second stage if they seemed to potentially address at least one of the identified policy-relevant research priorities pertaining to the HRH, health financing, role of the non-state sector, and ATM themes. We then obtained the abstracts of titles judged as potentially eligible.

In the second stage, we applied eligibility criteria to those abstracts and obtained the full texts of those judged as potentially eligible. In the third stage, we applied the eligibility criteria to the full texts. Records passed the first and the second screening if judged as potentially eligible by at least one of the two reviewers. For the third screening stage, the two reviewers compared results and resolved disagreements by discussion. When consensus could not be reached, a third reviewer made the final judgment.

### Data abstraction

One reviewer was responsible for filling the data extraction using a standardized and pilot-tested Excel form. Data were abstracted from two sources: the HSE record of the citation and the full text of the citation. The abstracted data from the HSE record consisted of the following: i) type of document: SR of effects or SR addressing other questions; ii) last year literature searched; iii) year of publication; iv) AMSTAR quality rating, when reported by HSE database; and v) countries in which primary studies were conducted when reported by HSE.

The abstracted data from the full-text consisted of the following: i) the source of publication: Cochrane Library or peer-reviewed journal; ii) the number of authors; iii) the number of authors affiliated with an institution based in the EMR; iv) whether the first author is affiliated with an institution based in the EMR; v) whether the corresponding author is affiliated with an institution based the EMR; vi) the number of included primary studies; vii) countries where included primary studies were conducted; viii) the number of included primary studies conducted in the EMR; ix) whether all included primary studies were conducted in the EMR.

### Classification of systematic reviews

Two reviewers classified, independently and in duplicate, each SR under the appropriate theme and sub-theme (see details below). They also matched SRs to the pre-identified policy-relevant research priorities. Results were compared and disagreements were resolved by discussion. When consensus could not be reached, a third reviewer made the final judgment.

The sub-themes were adapted from published frameworks and relevant literature. The sub-themes for HRH were adapted from two different frameworks: HRH action framework and the human resources for health common technical framework [17,18]. For the health financing, the sub-themes were derived from the Schieber et al. framework on health financing [19]. The ATM sub-themes were adapted from the WHO framework for ATM [20]. As for the role of the non-state sector, sub-themes were derived from a framework adapted from relevant literature [12,21,22]. Classification each SR under sub-themes helped in providing an understanding about the focus of SRs within each theme. It also allowed identification of knowledge gaps in terms of focus of SRs.

### Data analysis

We calculated Fleiss’ Kappa coefficient to measure agreement between reviewers for the abstract and full-text screening phases. Kappa statistics were calculated using the GraphPad Software. We used the following cut-offs to judge the degree of agreement: 0.21 to 0.40 for fair agreement, 0.41 to 0.60 for moderate agreement, 0.61 to 0.80 for substantial agreement, and 0.81 to 1.00 for almost perfect agreement [23,24]. We conducted descriptive analysis for the abstracted data using frequency and percentages.

## Results

**Figure 3:**
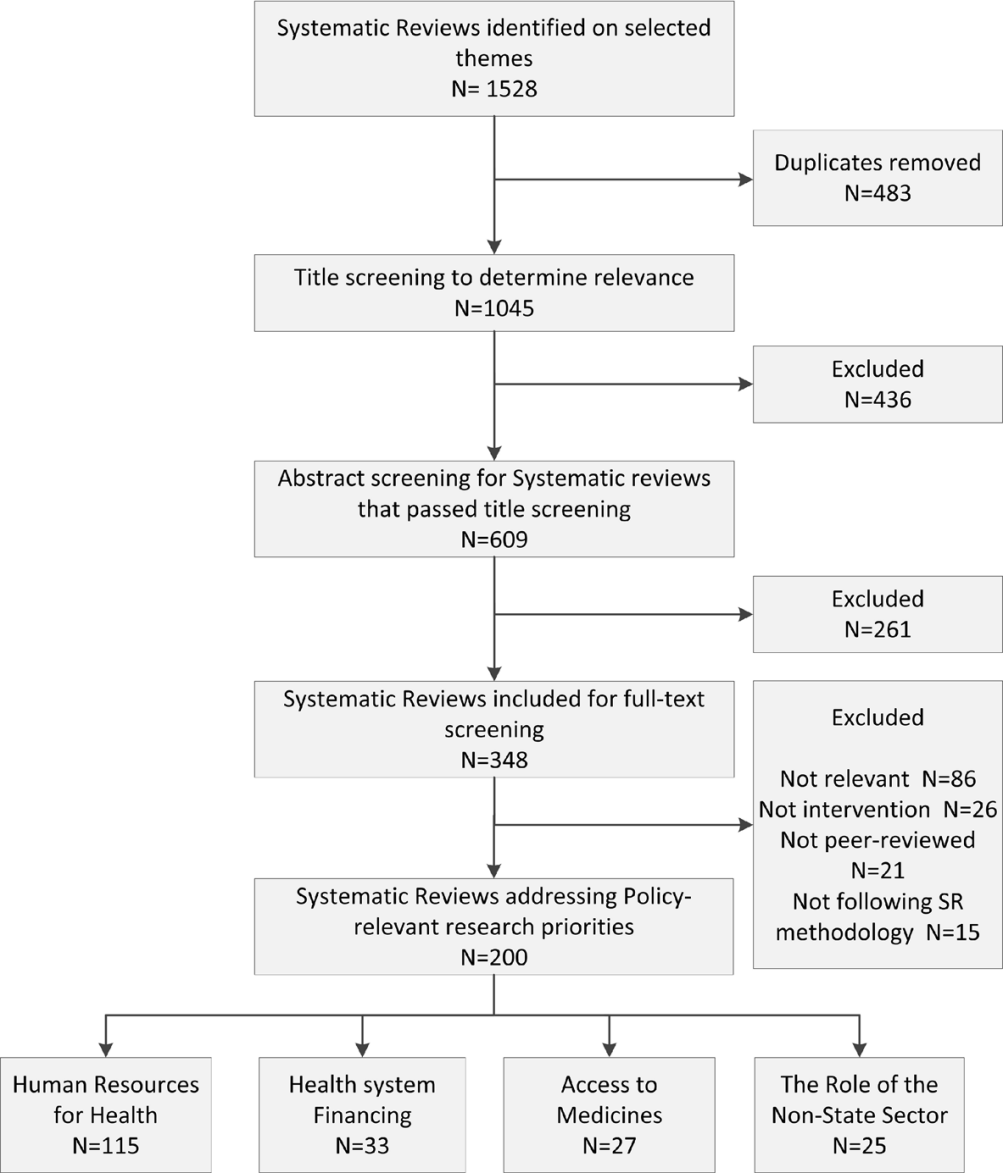
Selection process flowchart.

Out of the 1,045 SRs identified on selected themes and assessed for potential eligibility, a total of 200 SRs (19.1%) fit criteria for inclusion (Figure 3). The level of agreement between the two reviewers is considered to be good with Kappa equal to 0.72.

### Extent to which published SRs address EMR policy priorities and knowledge gaps

**Figure 4:**
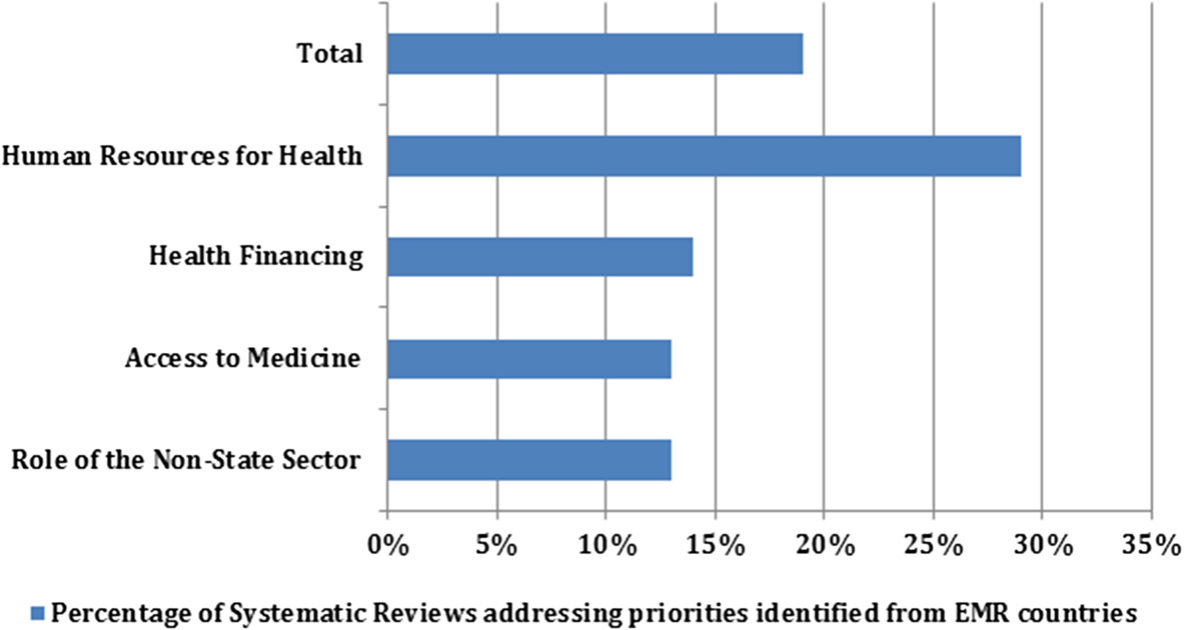
Extent to which the identified systematic reviews addressed EMR priorities by theme.

**Figure 5:**
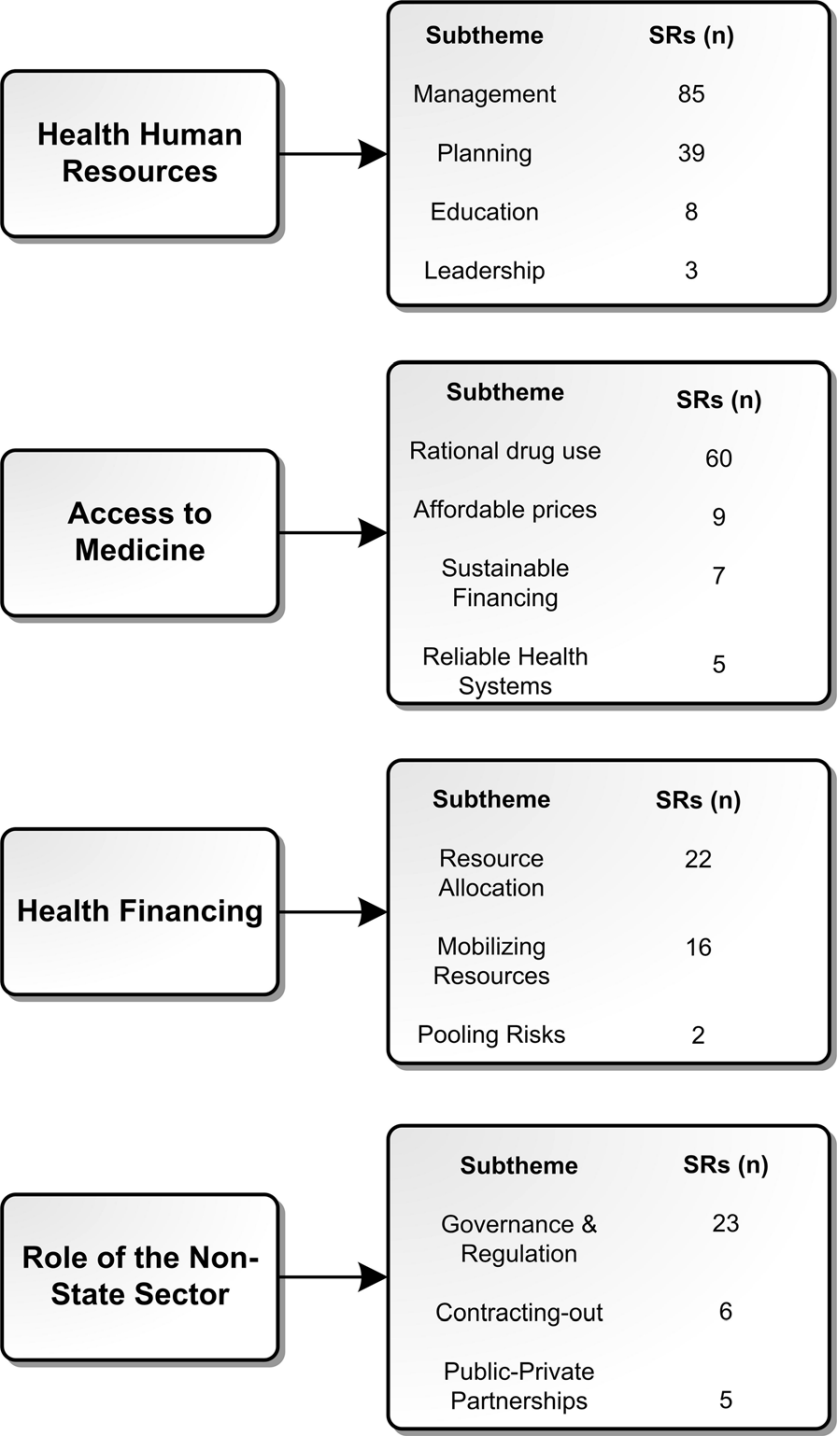
Themes stratified by sub-themes.

As indicated in Figure 3, the theme with the largest number of SRs included was HRH (115), followed by health financing (33), ATM (27), and role of the non-state sector (25).As indicated in Figure 4, 19.1% of the SRs identified from HSE addressed the priorities from the EMR. The HRH was the theme with the largest number of SRs addressing the priorities, 29% of the SRs focusing on HRH theme were eventually included; 13% of the SRs tackling the role of the non-state sector addressed the priorities. The quality of the included SRs is considered to be low. Out of the 147 SRs with reported AMSTAR quality rating, 110 SRs had a quality score below 8.Figure 5 shows the categorization of the included SRs into sub-themes. Within HRH, the sub-theme addressed by the largest number of SRs was “management” with 85 SRs. A low number of SRs focused on “leadership and education”. In terms of the ATM theme, most SRs focused on “rational drug use” and the fewest number of SRs were those addressing “reliable health systems”. In health financing, “resource allocation” was the focus of most SRs while only two SRs addressed “pooling risks”. In the role of the non-state sector, the “governance and regulation of the non-state sector” was the focus of most SRs within this theme.

Table 1 details the SRs addressing each of the policy-relevant priority pertaining to the four themes. Upon examining every theme on its own, many differences become clear (Table 1). The top two ranked policy priorities in HRH were not addressed by any SR. The priorities ranked 5 “Ways that can enable education and training programs to meet the population health needs” and 7 “Develop simulation models for HRH planning” in HRH were the focus of most SRs within this theme (26.1%). This is not the case for the health financing theme, whereby the top five priorities under this theme were addressed by SRs. Priorities ranked 6, 7, 8, and 9 had no SRs addressing them. The 2^nd^ ranked priority “household ability to pay for health care” was addressed with the highest number of SRs in this theme representing 48.4%. In ATM, the top ranked priorities had few SRs addressing them. For instance, 28.5% of the SRs addressed the first four priorities combined while 19% addressed the 7^th^ ranked priority alone. Most SRs in the ATM theme were focusing on 12^th^ ranked priority “alternative financing mechanisms to supplement public sector provision”. Although, the fewest number of included SRs were identified for the role of the non-state sector, these addressed the highest ranked priorities. The top priority, “ways to regulate and monitor the quality of care in the private sector” was the focus of most SRs within this theme (64%). The 2^nd^ and 3^rd^ priorities, “ways for the public and private sectors to complement their service delivery” and “ways to optimize the use of existing resources of the non-state sector to meet health system objectives” were addressed by 16% of the SRs. Priorities ranked 4 and 5 had no SRs addressing them.

### SR production in the region

**Figure 6:**
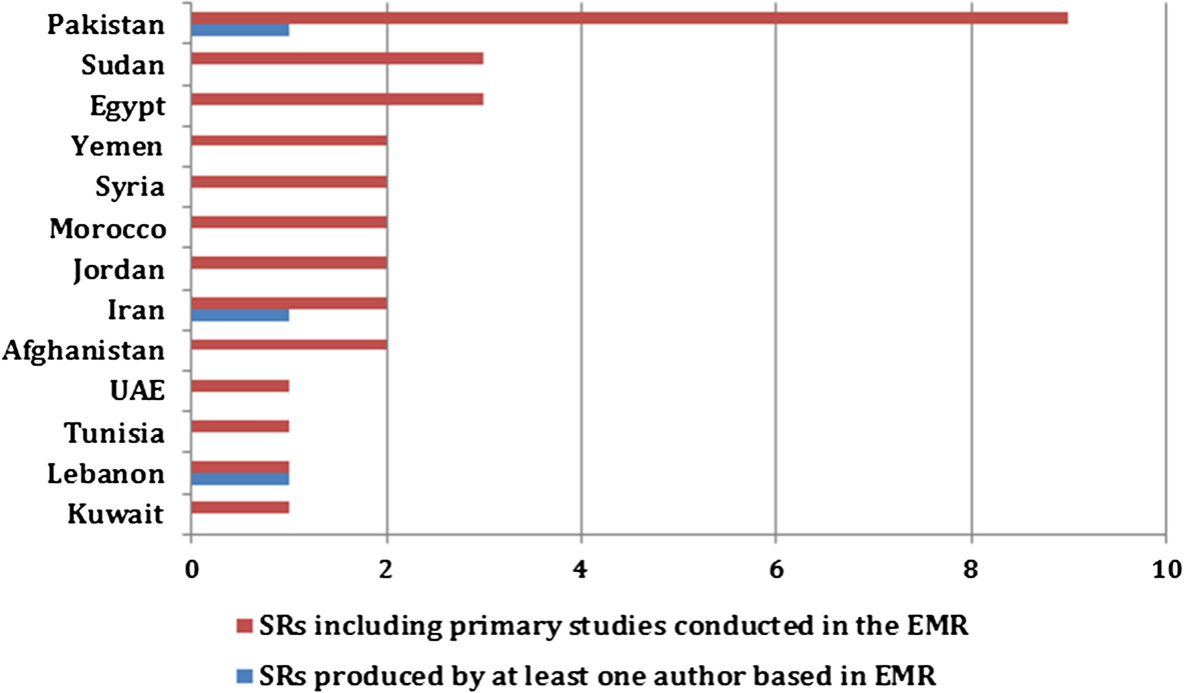
EMR countries with primary studies and authors contributing to systematic reviews.

A very low number of SRs was produced in the EMR (Table 2). Authors based in the region produced only three SRs addressing regional priorities (1.5%). However, those reviews were not focused on the EMR. The contributing authors were based in institutions from Lebanon, Pakistan, and Iran (Figure 6). Findings also showed low contribution of primary studies from the region to SRs with only 17 SRs (8.5%) benefiting from such studies. Studies from Pakistan were included in nine SRs while studies from Egypt were included in three. Eleven of the 24 EMR countries did not contribute any primary studies to SRs (Figure 5). The theme with the largest number of SRs including primary studies from the region was the role of the non-state sector with seven SRs out of the 17 SRs.

**Table 2:** State of production and contribution of the Eastern Mediterranean region (EMR) to systematic reviews (SRs)

**Parameter**	**n (%)**
Total SRs addressing policy-relevant research priorities	200 (19.1%)
SRs including primary studies conducted in the EMR	17 (8.5)
SRs produced by at least one author based in the EMR	3 (1.5)
SRs with EMR as the target jurisdiction	0 (0.0)

## Discussion

Study findings showed a gap in the production of SRs addressing policymakers’ and stakeholders’ priorities in the EMR. The distribution of SR production across the themes does not sufficiently correspond to priorities identified by policymakers and stakeholders in the EMR. Indeed, only 19.1% of the identified SRs matched those priorities. Moreover, these SRs matched lower ranked priorities. For instance, most of the identified SRs in HRH related to the management aspect while the EMR high ranked needs related to information systems for collaboration and coordination on a national level and identifying gaps in established education and training programs.

Several reasons contributed to the limited number of SRs that were matched to regional priorities. First, the state of production of reviews addressing broad health system and policy topics is already low [3]. Second, many of the priorities identified across the themes are context-specific such as “National database on non-state sector” or “Population health status and needs” and would only benefit from regionally-focused SRs rather than international ones. Most of the SRs identified were produced in high-income settings and, as such, are not expected to address the priorities of the EMR, which are probably very different. Finally, the priorities identified were policy-relevant priorities rather than questions for SRs of effectiveness. Many of the questions could be addressed by SRs of observational and qualitative studies, non-experimental studies, national health accounts, and single studies. This raises the need to develop a priority-setting tool that differentiates between policy priority questions that can be answered by SRs versus other types of evidence.

The study also shows the lack of SRs focusing on the EMR region as a target jurisdiction. This study identified no reviews addressing regional priorities with EMR as focus. A recent study shows that only 22% of the global stock of health policy research evidence was focused on LMICs [25]. This could also be linked to the lack of individual and institutional capacity. For instance, China and Brazil, with a large pool of review authors, were the focus of a higher number of SRs as compared to 41 other LMICs [5].

Another finding is the minimal production of SRs by authors based in the EMR. Out of 200 total SRs included, only three SRs were produced by authors based in the EMR. These findings come in line with prior studies assessing the global production of SRs. For instance, a study done by Law et al. [5], showed that authors based in the EMR collectively produced 195 SRs from 2003 to 2008 while authors based in China alone produced 575 SRs, and those in Brazil produced 395 SRs over the same period. This could be potentially related to issues of lack of resources and capacities on the institutional and individual levels [5]. An unpublished report by Bangpan et al. (2013) emphasized the lack of institutional capacity for SRs production and communication in LMICs in general and in the EMR in particular. The limited number of SRs produced from researchers in several countries in the region reflects limited capacity. The Cochrane collaboration, which has the most extensive network for SRs, has very few authors based in the EMR. In fact, with exception of Iran, Syria, and Egypt, all EMR countries have less than 16 Cochrane reviewers. Countries like Afghanistan and Sudan have only one Cochrane reviewer [personal communication with Bangpan et al., 2013]. This lack of individual and institutional capacity can highly affect the state of SR production. For instance, Iran, with the highest availability of Cochrane reviewers in the EMR (132), produced the highest number of SRs (67) over the period from 2003 to 2005 [personal communication with Bangpan et al., 2013; 5].

The low contribution of primary studies from the region to SRs was also highlighted in the study findings. A small percentage of SRs (8.5%) included studies from the EMR. This could be due to low production of single studies related to health systems and policy in the region or to the methodological quality of those studies. Findings corroborate a previous study from the region which reported limited production of health policy and systems research, in general, and the lack of those addressing priority issues in the EMR, in specific [26]. Interestingly, although the role of the non-state sector was the theme with the smallest number of identified SRs (n = 25), it was associated with the largest number of SRs utilizing primary studies from the region (7 SRs). This could reflect an interest in the role of the non-state sector in the EMR, especially since the role of public institutions is not as pronounced.Finally, the distribution of SR production within each theme was unequal among sub-themes and often swayed towards one sub-theme more than the other (Figure 5). For instance, in the ATM theme, although each sub-theme was representative of an equally important element of the framework, SRs tended to be focused on certain sub-themes more than others. This was also the case for the other three themes. This shows a gap in addressing certain topics in the literature. Some sub-themes are given more consideration than others in the literature, which can be explained by the fact that the global agenda is driven by the priorities of high income countries. This can also be interpreted by the challenges faced in some areas. For instance, the majority of SRs under the HRH theme focused on management while there is a gap in leadership. This probably reflects the challenges facing the HRH in the EMR specifically in terms of recruiting and retention.

### Strengths and limitations

To our knowledge, this situational analysis exercise is the first of its kind in the region in terms of matching policy priorities identified by policymakers and stakeholders with existing SRs. A second strength is the relevance of the themes addressed in this study to the EMR. The selection of themes of interest was based on empirical research identifying health policy priority needs of the region. The use of standard SR methodology in conducting the study also enhances its quality. This included a comprehensive search and duplicate processes for selecting studies and abstracting data. Moreover, the search was not limited by time or language, permitting the capture of a wider range of SRs pertaining to the priorities across the selected themes.

The study has a few limitations. The priorities identified in this paper stem from 11 LMICs of the EMR so they might not reflect the priorities in all EMR countries. However, given the similarities across the contexts of many countries in the region, the cross-cutting challenges and the high proportion of LMICs as compared to high-income countries in the region (more than 60%), these priorities are likely to reflect the situation in most EMR countries. The use of HSE as the only database might raise the possibility of missing certain reviews. However, HSE is a comprehensive and continuously updated database that draws its content from major sources of SRs. Additionally, limiting the inclusion criteria to SRs of effectiveness may have caused an underestimation of SRs addressing the priorities. Finally, this exercise focuses on four out of the six building blocks of a health system, as per WHO definition, and does not capture SRs addressing priorities pertaining to governance and health information. However, as demonstrated earlier, the selected four themes are of high priority in the region.

### Implications for policy and research

Findings from our study can inform researchers and research institutions in order to better align SR production with policy needs and priorities. Study findings can also inform funders to support the production of SRs to address gaps and policy priorities. Producing SRs that respond to policymakers’ priorities would increase the likelihood of the use of evidence generated in the policymaking process.

As the study shows a low production of SRs by authors based in the region, building capacities of researchers to conduct SRs on health policy and systems research could push towards the use of evidence in policymaking. Future studies examining the factors behind the low production of SRs in the EMR can also inform future work aiming at increasing SR production and promoting knowledge translation activities in the region. Building capacities of policymakers in evidence-informed policymaking and raising their awareness on the importance of SRs is crucial to increase the demand for SRs and the uptake of evidence into policies.

## Abbreviations

ATM: Access to medicine; EMR: Eastern Mediterranean region; HRH: Human resources for health; HSE: Health systems evidence; LMICs: Low- and middle-income countries; SRs: Systematic reviews; WHO: World Health Organization.

## Competing interests

The authors declare that they have no competing interests.

## Authors’ contributions

FE and EA made substantial contributions to the conception, design, analysis, and interpretation of results and write-up of the manuscript. LB and OK made substantial contributions to design, acquisition and review of systematic reviews, analysis of data, interpretation of results, and write-up of manuscript. CA contributed to acquisition and review of systematic reviews and to write up of the manuscript. RF and RH contributed to the review of systematic reviews and analysis of data. All authors read and approved the final manuscript.

## Supplementary Material

Additional file 1 **Search Strategy.** The file contains detailed search terms used for each theme. Click here for file
