# Supplementary material for: Systematic reviews addressing identified health policy priorities in Eastern Mediterranean countries: a situational analysis
Source: Health Res Policy Syst. 2014 Aug 20;12:48. doi: 10.1186/1478-4505-12-48 (PMC4237801; doi:10.1186/1478-4505-12-48)
Supplement: Additional file 1 — Search Strategy. The file contains detailed search terms used for each theme. [file 1478-4505-12-48-S1.docx]

# Additional file 1: Search Strategy

## Human Resources for Health

*Search terms obtained from the HSE database*

| By whom care is provided | System - Need, demand & supply |
| --- | --- |
|  | System - Recruitment, retention & transitions |
|  | System - Performance management |
|  | Workplace conditions – Provider satisfaction |
|  | Workplace conditions – Health & safety |
|  | Skill mix – Role performance |
|  | Skill mix – Role expansion or extension |
|  | Skill mix - Substitution |
|  | Skill mix - Multidisciplinary teams |
|  | Skill mix – Communication & case discussion between distant health professionals |
|  | Staff – Training |
|  | Staff - Support |
|  | Staff - Workload/workflow/intensity |

## Health Financing

*Search terms obtained from HSE database*

| Financing systems | Taxation |
| --- | --- |
|  | Social health insurance |
|  | Community-based health insurance |
|  | Community loan funds |
|  | Private insurance |
|  | Health savings accounts (Individually financed) |
|  | User fees |
|  | Donor contributions |
| Funding organizations | Fee-for-service |
|  | Capitation |
|  | Global budget |
|  | Prospective payment (for a particular diagnosis, product, etc.) |
|  | Indicative budgets |
|  | Targeted payments / penalties |
| Remuneration | Fee-for-service |
|  | Capitation |
|  | Salary |
|  | Prospective payment |
|  | Fundholding |
|  | Indicative budgets |
|  | Targeted payments / penalties |
| Incentivizing consumers | Premium |
|  | Cost-sharing (e.g., co-payment, user fee) |
|  | Health Savings accounts |
|  | Targeted payments (e.g., conditional cash transfer)/ penalties |

| Purchasing products & services | Scope & nature of insurance plans |
| --- | --- |
|  | Lists of covered / reimbursed products & services (e.g., “positive” lists such as formularies & “negative” lists such as restrictions) |
|  | Lists of substitutable products & services |
|  | Restrictions in coverage / reimbursement rates for covered products & services |
|  | Caps on coverage / reimbursement for covered products & services |
|  | Prior approval requirement |

## Access to medicine

*Search terms obtained from HSE database*

| Commercial authority | Licensure / registration |
| --- | --- |
|  | Patents & profits |
|  | Pricing & purchasing |
|  | Marketing |
|  | Sales & dispensing |
|  | Commercial liability |
| Purchasing products & services | Scope & nature of insurance plans |
|  | Lists of covered/reimbursed organizations, providers, services & products |
|  | Restrictions in coverage/reimbursement rates for organizations, providers, services & products |
|  | Caps on coverage/reimbursement for organizations, providers, services & products |
|  | Prior approval requirements for organizations, providers, services & products |
|  | Lists of substitutable services & products |

*Additional search terms found relevant by the research team (WHO, 2010)*

| Commercial Authority | Utilization OR Utilisation |
| --- | --- |
| Purchasing products and services | Pharmaceutical* |
| Manufacturing | Drug* |
| Registration | Medicines |
| Selection | Medication |
| Pric* | procurement |
| Distribution | Promotion |
| Inspection | Marketing |
| Prescrib* | Access |
| Dispens* | Access to medicines |
| drug OR medication OR medicine | Quality and medicines/medication/drug/pharmaceutical |
| drug OR medication OR medicine AND access | counterfeit medicines/medication/drug/pharmaceutical |
| drug OR medication OR medicine AND afford* | Generic AND/OR brand |
| drug AND availab* OR medication AND availab* OR medicine AND availab* |  |

* refers to truncation of search terms

## The Role of the Non-State Sector

*Search terms obtained from HSE database*

| Policy authority | Stewardship of the non-state sector’s role in financing & delivering |
| --- | --- |
|  | Stakeholder participation in policy & organizational decisions |

*Additional search terms developed by the research team based on relevant literature (Patouillared et al. 2007; El-Jardali et al. 2011; Green 1987).*

| Private sector | Government |
| --- | --- |
| Civil society | Government regulation |
| Informal sector | Dual practice |
| Non-governmental organization | Health service performance agreement |
| Faith-based organization | Pay-for-performance |
| Community group | Quality care |
| Public-private partnership | Standardized and indicators |
| Accreditation | Standardized or indicators |
| Contracting | Balanced score card |
| Public reporting | Sector |
